# Water use practices, water quality, and households’ diarrheal encounters in communities along the Boro-Thamalakane-Boteti river system, Northern Botswana

**DOI:** 10.1186/s41043-015-0031-z

**Published:** 2015-11-18

**Authors:** G. Tubatsi, M. C. Bonyongo, M. Gondwe

**Affiliations:** Okavango Research Institute, University of Botswana, Private Bag 285, Maun, Botswana

**Keywords:** Household, Hygiene, Ngamiland, Okavango Delta, Water storage, Water treatment, Waterborne diseases

## Abstract

**Background:**

Some rural African communities residing along rivers use the untreated river water for domestic purposes, making them vulnerable to waterborne diseases such as diarrhea.

**Methods:**

We determined water use practices and water quality, relating them to prevalence of diarrhea in communities along the Boro-Thamalakane-Boteti river system, northern Botswana. A total of 452 households were interviewed and 196 water samples collected show during February, May, September, and December 2012 in settlements of Boro, Maun, Xobe, Samedupi, Chanoga, and Motopi. Information was sought on water use practices (collection, storage, and handling) and diarrheal experience using questionnaires. Water quality was assessed for physicochemical and microbiological parameters using portable field meters and laboratory analysis, respectively.

**Results:**

All (100 %) of the river water samples collected were fecally contaminated and unsuitable for domestic use without prior treatment. Samples had Escherichia coli (*E.coli*) and fecal streptococci levels reaching up to 186 and 140 CFU/100 ml, respectively. Study revealed high dependence on the fecally contaminated river water with low uptake of water treatment techniques. Up to 48 % of households indicated that they experience diarrhea, with most cases occurring during the early flooding season (May). Nonetheless, there was no significant relationship between river water quality and households’ diarrheal experience across studied settlements (*p* > 0.05). Failure to treat river water before use was a significant predictor of diarrhea (*p* = 0.028).

**Conclusions:**

Even though the river water was unsafe for domestic use, results imply further recontamination of water at household level highlighting the need for simple and affordable household water treatment techniques.

## Background

Diarrhea remains one of the leading causes of morbidity and mortality worldwide [1–3] despite improved health technologies, management, and increased use of oral rehydration therapy [4, 5]. Worldwide, about two billion cases of diarrheal diseases are registered annually, out of which 1.9 million children under the age of 5 die particularly in developing countries. Diarrheal diseases display distinct geographical variation and seasonality [5–8] due to varying occurrence of their etiological agents in the environment [9]. Several authors [10–13] attributed the burden of diarrheal diseases to the environment and associated risk factors, particularly unsafe drinking water from open water sources, poor sanitation, and poor hygiene. While causes of diarrheal diseases are multi factorial, the use of untreated water harboring diarrheal pathogens [10] remains a significant contributor to most outbreaks [11, 12].

Rotavirus and *Escherichia coli* have been implicated for most diarrheal outbreaks in developing countries [6, 13, 14] where some communities use water of quality lower than the recommended standards. When contaminated water is ingested, pathogens invade the intestines’ epithermal walls, disturbing mechanisms that transport water and electrolytes and causing diarrhea [15]. Therefore, higher microbial concentrations in water increase chances of invasion hence diarrhea.

The revised standards [16] for drinking water recommend that the water should be free from pathogens and microbial indicators, pH between 6 and 8, turbidity less than 5 nephelometric turbidity units (NTU), and conductivity less than 1000 μS/cm. Microbial water quality indicators such as coliforms, fecal streptococci, and *E. coli* are often used to assess water quality despite the fact that they are not responsible for causing diarrhea. In addition to the quality of water at source point, the safety of drinking water and the health risks associated with its use thereof can further be influenced by hygiene practices during transportation, storage, and handling leading to microbiological contamination [4, 17–19]. Recent studies demonstrated that uptake of water treatment techniques at household level such as boiling, chlorination, and filtration could improve drinking water quality and significantly reduce the burden of diarrhea [1, 20, 21].

Like in other sub-Saharan countries, diarrhea remains a major public health concern in Botswana, particularly among children [22] despite strengthened surveillance and launching of programs such as Integrated Management of Childhood Illnesses (IMCI). Although 95 % of Botswana’s population is reported to have access to treated safe water supply [23], dependency on untreated open water sources in ungazetted rural communities presents serious health risks. Unlike in gazette settlements where the government provides most of the services, water supply and many other services in ungazetted settlements are the responsibility of individual households. As such, outbreaks of water-related diseases like diarrhea are rampant mostly in the Ngamiland district in the northern part of the country, where many settlements are ungazetted and have ample access to open surface water from the Okavango Delta and its associated tributaries. For example, records at Letsholathebe II referral hospital in Maun, the capital town of Ngamiland district, show that 120 cases of diarrhea were registered over a 2-week period in June 2012. On the other hand, some landuse activities along the Thamalakane-Boteti river such as residential areas, lodges, hotels, and grazing have been reported to negatively impact on the water quality [24, 25], further pointing out to the potential health risks associated with water in these river systems.

Currently, the level of dependency on water from open sources, water use practices, and degree to which the quality of water contributes to diarrheal prevalence in Botswana has not been explored extensively. We assessed river water quality and water use patterns in selected communities along the Boro-Thamalakane-Boteti river system, an outlet of the Okavango Delta in the northern Botswana to establish their potential contribution to the prevalence of diarrheal diseases. Specifically, we (i) established the water use practices of selected respondents, (ii) related the occurrence of diarrheal diseases outbreaks to water use practice in the selected communities at different flooding seasons, and (iii) relate the occurrence of diarrheal diseases to selected water quality indicators. We hypothesized that (i) the occurrence of diarrhea will increase with increased dependency on river water, (ii) the occurrence of diarrhea will increase with low uptake of water treatment in households, and (iii) the occurrence of diarrhea in a settlement will increase as river water quality declines.

## Methods

### Study sites

**Fig. 1:**
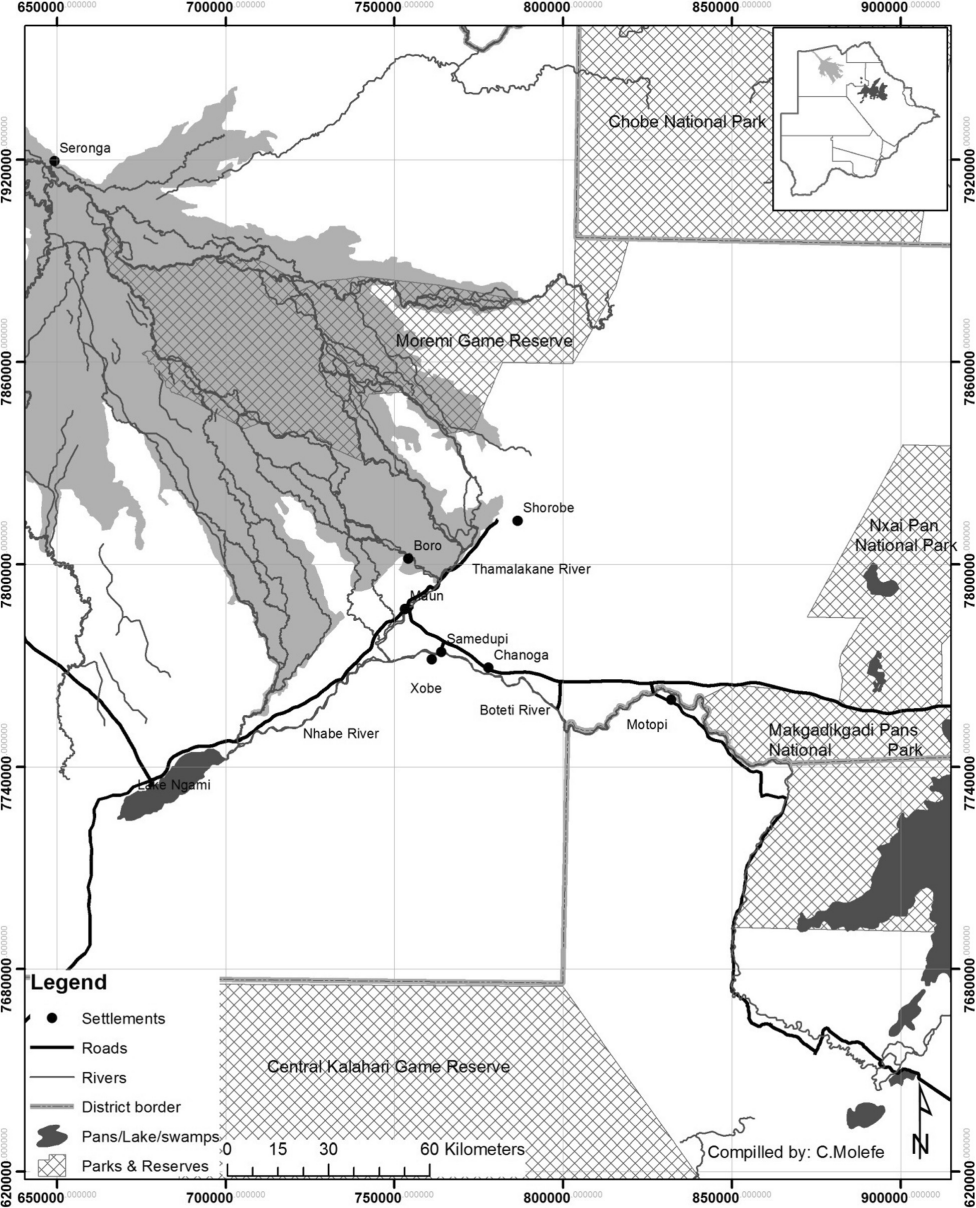
Map showing selected study sites along the Boro-Thamalakane-Boteti river system

Botswana (582,000 km^2^) is a semi-arid, land-locked country in southern Africa. Rainfall occurs mostly between November and March and is highly unreliable, ranging from 310 to about 580 mm year^−1^. The current study was conducted in settlements of Boro, Maun, Xobe, Samedupi, Chanoga, and Motopi along a 100-km stretch on the Boro-Thamalakane-Boteti (BTB) river system between the Okavango Delta and Makgadikgadi Pans (Fig. 1). Boro and Thamalakane rivers which drain the opposite sides of the Chief’s Island in the Delta join a few kilometers upstream of Maun but bifurcate further downstream to form the Nhabe and Boteti rivers which drain into Lake Ngami on the south-west and the Makgadikgadi salt Pans to the south-east, respectively (Fig. 1). Livelihood activities in the study settlements are diverse and vary seasonally and include livestock and arable farming, fishing, safari (especially Mokolo poling at Boro), and sale of crafts and natural products or veld products [26]. A few residents work in government facilities, safari companies, and trusts or employed under the government funded “Ipelegeng” (*self-reliance*) scheme (Table 1).

**Table 1:** Characteristics of settlements sampled for the study. Number of households was estimated by dividing total population of each settlement by the average household size of 5.4 reported by CSO (2010) for the Ngamiland district

Settlement	Distance (km)	Population	Number of households	Type of settlement
Boro	−11	393	73	Ungazetted
Maun	0	2053^a^	380	Gazetted
Xobe	13	260	48	Ungazetted
Samedupi	20	471	87	Ungazetted
Chanoga	30	347	64	Ungazetted
Motopi	87	3591	248	Gazetted

^a^Note that the population for Maun indicated here is only for Shashe ward and Matlapana which were sampled for this study. The total population of Maun is 60, 273 (CSO, 2012)

### Sampling

The study was conducted in 2012 during four distinct flooding seasons in the BTB river system; the late flood recession season (February), early flooding season (May), peak flooding season (September), and early flood recession season (December). A semi-structured questionnaire was developed, pre-tested in Matlapaneng (a ward in Maun) and Xobe, and rectified accordingly. The questionnaire was administered through face-to-face interviews in the local language (Setswana). We collected information from a total of 452 randomly selected households in the selected settlements on sources of water for domestic chores, household dependence on river water, uses of river water, their water storage practices, and water treatment techniques known and/or used in their households. Respondents were also asked about their encounters and experiences with diarrhea in their households. Diarrhea was defined to each respondent as passing out three or more watery stools within a period of 24 h. Respondents were those members of households who were responsible for general household chores including fetching water.

### Socio-economic characteristics of respondents

Majority (405) of the respondents were females, while only few (47) were males. A quarter (113) of respondents were aged below 30 years; while almost half (242) were between 31 and 45 years, 81 respondents were between 46 and 65 years, and just a small number (16) aged over 65 years. Majority (316) of the respondents were not employed, 52 employed in the government sector, and 84 were self-employed or in non-governmental employment. Only 86 of the respondents reported a monthly income over P500, and majority (366) were earning P500 or less per month. Almost a third (127) of the total respondents did not have formal education. Majority had attained primary (128) and Junior Secondary (134) education. Thirty six (36) have attained senior secondary education. Very few respondents (13) have attained up to tertiary education level.

### Ethical considerations

A written ethical clearance to conduct the study was obtained from the Health Research and Development Division, Ministry of Health (Gaborone, Botswana). Participation by respondents in the study was entirely on voluntary basis. The study objectives, procedure, and benefits were clearly defined to potential respondents; after which, those who agreed to participate in the study were asked to sign the informed consent which was sought before participants took part in the study. Participation was entirely on voluntary basis. Participants who were not willing to take part in the study were allowed to do so without giving any reasons for their decisions. Information obtained during interviews was kept confidential and anonymous and was used only in this study.

### Water quality analysis

All the six study settlements were sampled once in February, May, September, and December 2012. These months corresponded to different seasons in relation to flooding (February is late flood recession season; May is early flooding season; September is peak flooding season and December is early flood recession). At each sampling point, in situ water temperature (°C), pH, dissolved oxygen (DO, mg l^−1^ ), electrical conductivity (EC, μS cm^−1^), and turbidity (NTU) were measured using WTW pH 330i, WTW OXi 330i, WTW Cond 720, and TN-100 portable Turbidimeter meters, respectively.

A total of 196 water samples were collected in 250-ml borosilicate glass bottles sterilized at 121 °C for 15 min. The samples were transported to the laboratory at the Department of Water Affairs in Maun in cooler boxes with ice. At the laboratory, water samples were analyzed for fecal coliforms, *E. coli*, and fecal streptococci using membrane filter technique as described in [27]. Aliquots of the water samples (100 ml) were filtered through sterile 0.45-μm membrane filter papers which were incubated in agar plates at 37 °C for coliforms; *E. coli* was incubated at 35 °C and fecal streptococci at 44.5 °C. Oxoid CM 1031 Membrane Lactose Glucoronide Agar (MLGA) was used for identification and enumeration of *E. coli* and coliforms while Oxoid CM 0377 Slanetz & Bartley medium was used for isolation of fecal streptococci.

### Data analyses

Data were captured using Statistical Package for Social Scientists (SPSS) version 20.0. Frequencies and percentages were used for determining water use practices and households’ diarrheal encounters. Analysis of variance (ANOVA) was used to compare households’ diarrheal encounters’ means across study sites and seasons. Pair wise comparisons of households’ diarrheal encounters between sites and seasons were computed using Turkey’s honest significant difference (HSD) test. Correlations between water use practices, water quality, and households’ diarrheal encounters were measured before regression analysis was done. Multiple regression analyses were used to measure associations at 95 % confidence level. Further analyses were computed using SYSTAT 13 statistical software (SYSTAT Inc.).

## Results

### Water sources

All (100 %) respondents in the ungazetted settlements of Xobe and Samedupi fetched their water from the river for all domestic purposes. In contrast, settlements of gazetted Boro, Maun, Chanoga, and Motopi had safe water supply systems providing potable water to residents. All (100 %) interviewed households in Boro, Chanoga, and Motopi, and 70 % in Maun, indicated they sometimes get water from the river at least once in a week for domestic purposes despite available connections to treated water supplies through private and communal standpipes.

Only a few respondents (1.7 % in Motopi) indicated harvesting rain water from corrugated roofs during the rainy season. The low application of rainwater harvesting technique was confirmed by a quick survey of the houses in the settlements which showed high numbers of traditional grass thatched huts particularly in all the studied settlements except Maun. None of the respondents in Maun had indicated ever harvesting rain water.

### Water collection and storage

**Fig. 2:**
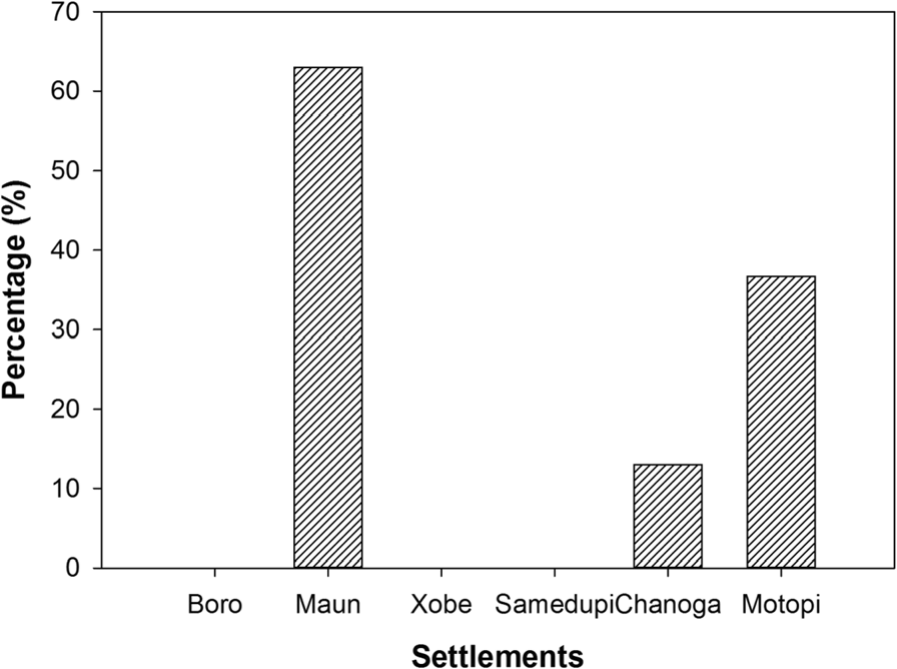
Percentage of interviewed households with standpipes at home in settlements along the Boro-Thamalakane-Boteti river system

Almost two thirds (63 %) of interviewed households in Maun had piped/tapped water connections in their homes (Fig. 2). The percentages were lower for Motopi (36.7 %) and Chanoga (13 %). Those without water connections at home use communal standpipes, which are centrally located to be used by all residents and supplied by the government through the Department of Water Affairs. Boro, Xobe, and Samedupi did not have access to treated water supply altogether and collected their water from the river.

**Fig. 3:**
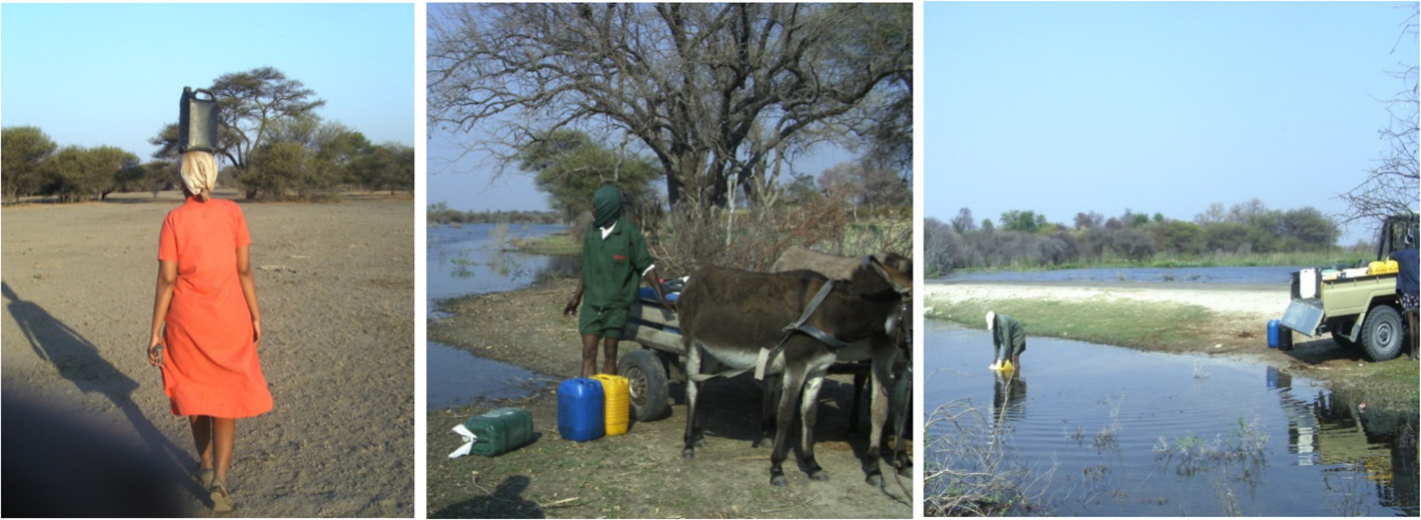
Some of the water fetching practices in selected settlements along the Boro-Thamalakane-Boteti river system, Ngamiland District, Botswana

Water fetching patterns were observed to be similar in all the study settlements from sources (river, taps) which were generally less than 2 km from households and stored in containers of various sizes such as buckets, 25-l jerry cans (locally called “Dikupu”), 200-l drums, and 260-l or more polyethylene tanks manufactured by JOJO Tanks (Pty) Ltd, South Africa (Fig. 5). While households in gazetted settlements are generally within 400 m from communal water standpipes, residents in gazetted settlements had to travel longer distances to fetch water. Water from source was transported home either on the head by female individuals, donkey carts, and cars by male household members (Fig. 3). Because large storage containers were usually used and water was sparely used by household members, the stored water took a several days to finish, and as result, the containers were not regularly cleaned before refilling. It was also observed that not all containers had lids, and in some instances, the lids were not of the right size and therefore did not fit properly.

### River water usage

**Fig. 4:**
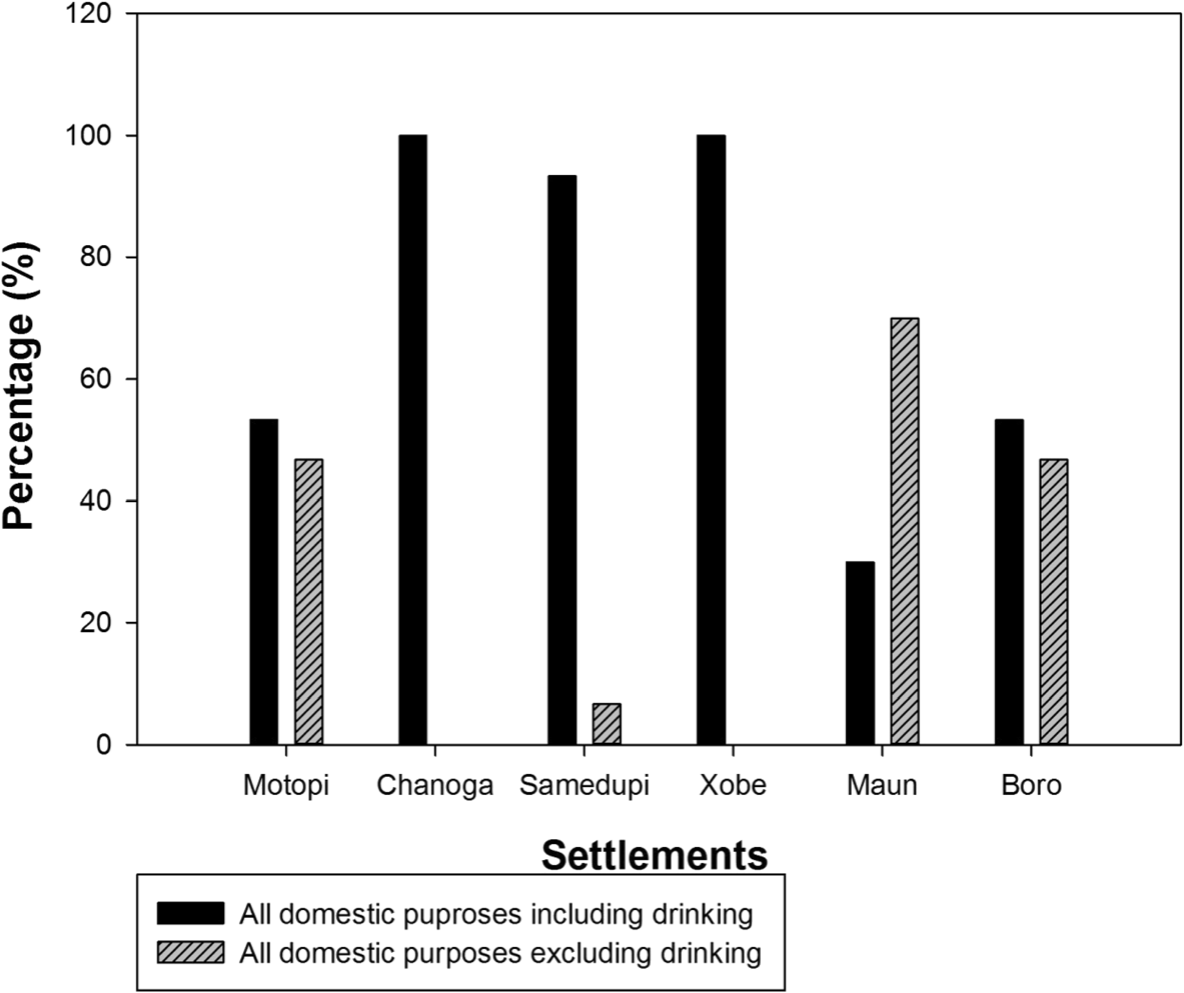
Domestic uses of river water for communities along the Boro-Thamalakane-Boteti river system

The river water was used for drinking and non-drinking purposes such as cooking, washing, bathing, and watering gardens. Almost all of respondents (>90 %) in Xobe, Samedupi, and Chanoga indicated using river water for all domestic purposes including drinking compared to 30 % in Maun and slightly above half (53.3 %) of the households interviewed in Boro and Motopi (Fig. 4).

### Water treatment

Boiling was the only form of water treatment known by most respondents, especially for drinking water. However, few respondents reported boiling river water for drinking in Boro (12.8 %), Maun (20.4 %), Xobe (14.9 %), Samedupi (15.0 %), Chanoga (24.4 %), and Motopi (25.9 %). Respondents further indicated that they boiled drinking water particularly for children below the age of 5 only. The rest of the household members frequently consumed untreated river water. Some of the reasons given for not treating river water before drinking included (i) water loses taste or tastes unpalatable after boiling, (ii) it takes time to boil and cool water for drinking while one is thirsty, (iii) boiling reduces the amount of water, (iv) boiling water for large families is tedious (iv), the river water is generally perceived to be clean and with minimal health risks to consumers, and (v) residents feel they have gained immunity against waterborne diseases (including diarrhea) associated with the consumption of river water.

### Physicochemical and microbiological quality of the river water

The quality of water in the Boro-Thamalakane-Boteti river system reflected by physicochemical and microbiological parameters was unsuitable for domestic use as turbidity, *E. coli*, and fecal streptococci concentrations exceeded recommended limits for drinking water set by the Botswana Bureau of Standards (Table 2). We noted a significant decline in water quality from settlements upstream of urban Maun (Boro) towards downstream (Xobe, Samedupi, Chanoga, and Motopi). The quality of water in the river system improved as the water level in the system increased during the peak flooding season (September) and declined subsequently thereafter.

**Table 2:** Water quality indicators (mean ± sd) for LFR, EF, PF, and EFR seasons in the BTB river system

	Settlements						
	Boro	Maun	Xobe	Samedupi	Chanoga	Motopi	BOBS
Temperature (°C)							
LFR	26.5 ± 0.1	30.3 ± 0.0	32.0 ± 0.1	28.8 ± 0.1	32.1 ± 0.0	30.6 ± 0.0	N/A
EF	19.5 ± 0.2	20.6 ± 0.0	17.8 ± 0.0	19.5 ± 0.0	19.2 ± 0.0	18.8 ± 0.0	
PF	20.9 ± 0.0	23.2 ± 0.0	21.8 ± 0.1	21.5 ± 0.0	24.2 ± 0.0	22.8 ± 0.0	
EFR	26.4 ± 0.0	27.5 ± 0.1	23.7 ± 0.0	24.6 ± 0.2	29.2 ± 0.1	28.4 ± 0.0	
Conductivity (μS cm^-1^)							
LFR	118.2 ± 0.6	123.0 ± 0.7	123.2 ± 0.8	127.4 ± 0.1	134.0 ± 0.0	131.1 ± 0.1	<1000
EF	104.0 ± 1.2	121.6 ± 9.3	124.1 ± 0.3	126.4 ± 0.1	133.0 ± 0.0	152.7 ± 0.8	
PF	92.1 ± 0.8	113.3 ± 1.8	104.1 ± 0.0	110.0 ± 0.1	106.6 ± 2.2	112.8 ± 7.9	
EFR	113.8 ± 0.5	122.1 ± 0.1	126.1 ± 1.0	127.8 ± 0.0	128.3 ± 0.1	119.4 ± 0.1	
pH							
LFR	6.7 ± 0.0	7.2 ± 0.1	7.8 ± 0.4	7.4 ± 0.0	8.1 ± 0.0	7.6 ± 0.2	6.5–8
EF	6.8 ± 0.0	7.4 ± 0.3	7.2 ± 0.1	7.5 ± 0.1	7.6 ± 0.0	7.6 ± 0.0	
PF	7.3 ± 0.0	7.4 ± 0.2	7.5 ± 0.1	7.3 ± 0.0	7.7 ± 0.1	7.4 ± 0.0	
EFR	7.0 ± 0.0	7.1 ± 0.0	7.5 ± 0.2	7.3 ± 0.0	7.8 ± 0.0	6.6 ± 0.1	
Turbidity (NTU)							
LFR	2.4 ± 0.2	6.8 ± 0.2	11.4 ± 0.20	4.1 ± 0.2	4.4 ± 0.4	8.8 ± 0.2	5
EF	0.8 ± 0.0	3.0 ± 0.2	4.9 ± 0.7	4.8 ± 0.2	3.3 ± 0.4	10.0 ± 0.5	
PF	1.7 ± 0.2	3.3 ± 0.6	8.0 ± 0.2	3.8 ± 0.2	8.0 ± 0.6	9.0 ± 0.1	
EFR	1.3 ± 0.0	6.3 ± 0.2	3.7 ± 0.7	1.9 ± 0.2	2.9 ± 0.2	8.1 ± 0.7	
*E. coli* (CFU/100 ml)							
LFR	54 ± 1	126 ± 0	127 ± 2	64 ± 4	82 ± 5	98 ± 15	0
EF	6 ± 3	168 ± 1	93 ± 2	186 ± 5	77 ± 2	139 ± 4	
PF	22 ± 0	102 ± 1	70 ± 1	106 ± 3	43 ± 3	32 ± 1	
EFR	20 ± 1	123 ± 1	56 ± 1	84 ± 2	40 ± 4	104 ± 9	
Fecal streptococci (CFU/100 ml)							
LFR	67 ± 8	140 ± 11	76 ± 6	93 ± 6	89 ± 3	26 ± 1	0
EF	2 ± 0	66 ± 8	30 ± 4	80 ± 1	21 ± 0	46 ± 2	
PF	12 ± 0	47 ± 1	4 ± 1	21 ± 0	47 ± 2	21 ± 2	
EFR	6 ± 0	53 ± 2	21 ± 4	37 ± 1	54 ± 8	32 ± 1	

### Households’ diarrheal encounters

**Fig. 5:**
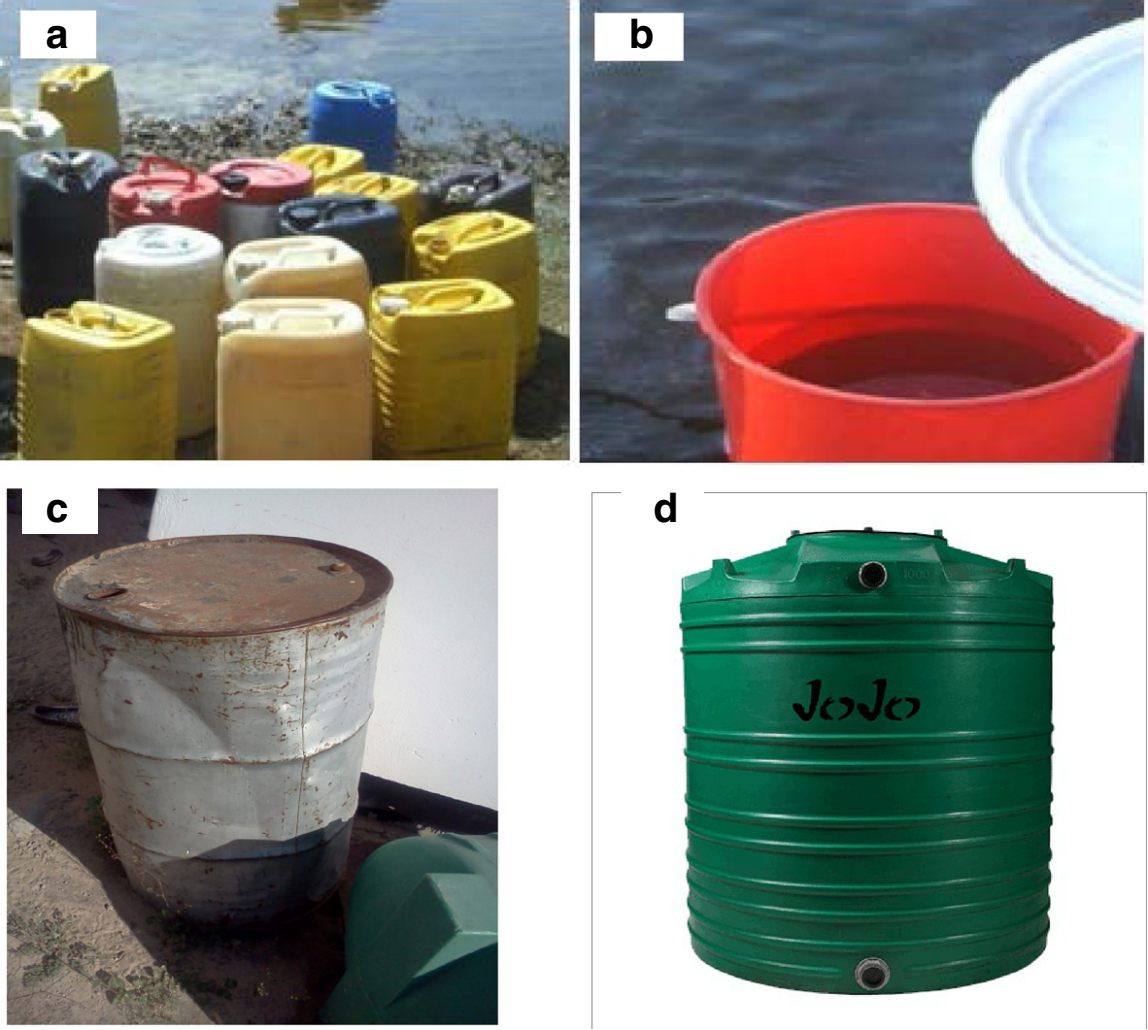
Some of the containers used by residents in the selected settlements to fetch and store water in households for their domestic uses—**a** jerry cans, **b** bucket, **c** drums, **d** polyethylene containers (*Jojo*)

Diarrhea cases varied across settlements (Fig. 5). Generally, respondents in Boro (upstream settlement of Maun) and Maun residents reported less diarrhea cases than downstream settlements of Xobe, Samedupi, Chanoga, and Motopi. During the early flooding season (May), about 33 % of the respondents in Boro reported diarrhea cases compared to 38–66 % settlements downstream of Maun reported cases of diarrhea (Fig. 5b). A similar pattern where upstream had less prevalence than downstream settlements was noted again during the peak flood season (September) (Fig. 5c). Prevalence recorded in Maun during the early (18 %) and late (13 %) flood recession seasons was lower compared to what was recorded in downstream settlements of Xobe, Samedupi, Chanoga, and Motopi (Fig. 5a, d). Downstream settlements recorded in 27–38 % and 20–27 % of the interviewed households during the early and late flood recession seasons, respectively. A similar pattern where prevalence increased in downstream settlements was observed again during the peak flooding season (September) (Fig. 5c).

**Fig. 6:**
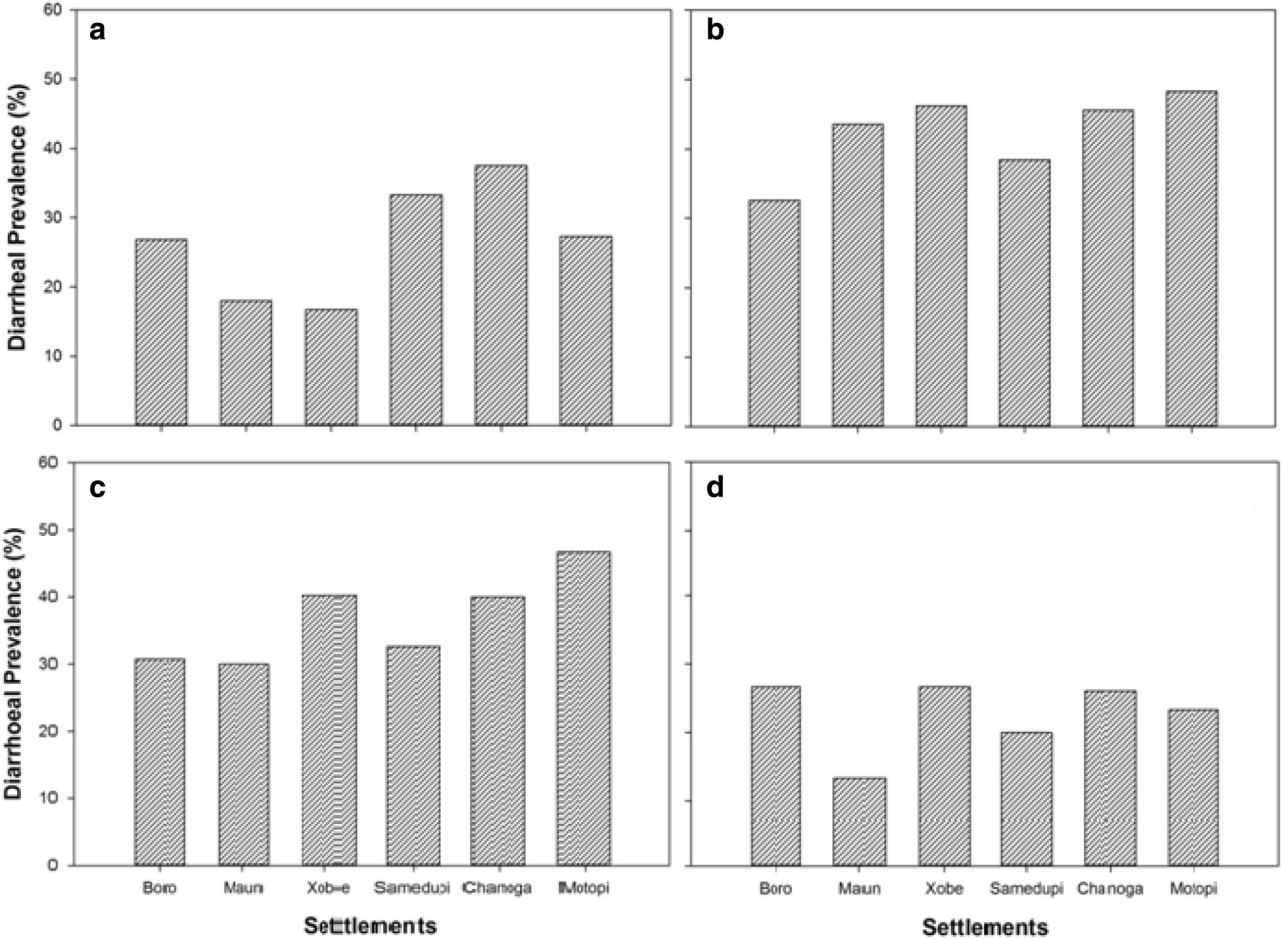
Spatial variation of diarrheal for **a** late flood recession season, **b** early flooding season, **c** peak flooding season, and **d** early flooding season among 160 communities along the Boro-Thamalakane-Boteti river system. Seasons *LFR* late flooding recession, *EF* early flooding, *PF* peak flooding, *EFR* early flood recession

A comparison of diarrhea cases within each settlement for the entire study period (February to December 2012) revealed a similar pattern (Fig. 6). All settlements recorded the highest number of cases during the early flooding season (May) which dropped in the subsequent seasons. However, respondents in Boro reported little variation in the number of diarrhea cases across seasons with a range of 5.9 % (SD = 2.93) compared to Maun and downstream settlements; Maun (30.3 %, SD = 13.6), Xobe (29.5 %, SD = 13.6), Samedupi (18.4 %, SD = 7.82), Chanoga (19.5 %, SD = 8.20), and Motopi (25.0 %, SD = 12.9) (Fig. 6a–f). Changes in prevalence were more profound in Maun.

**Fig. 7:**
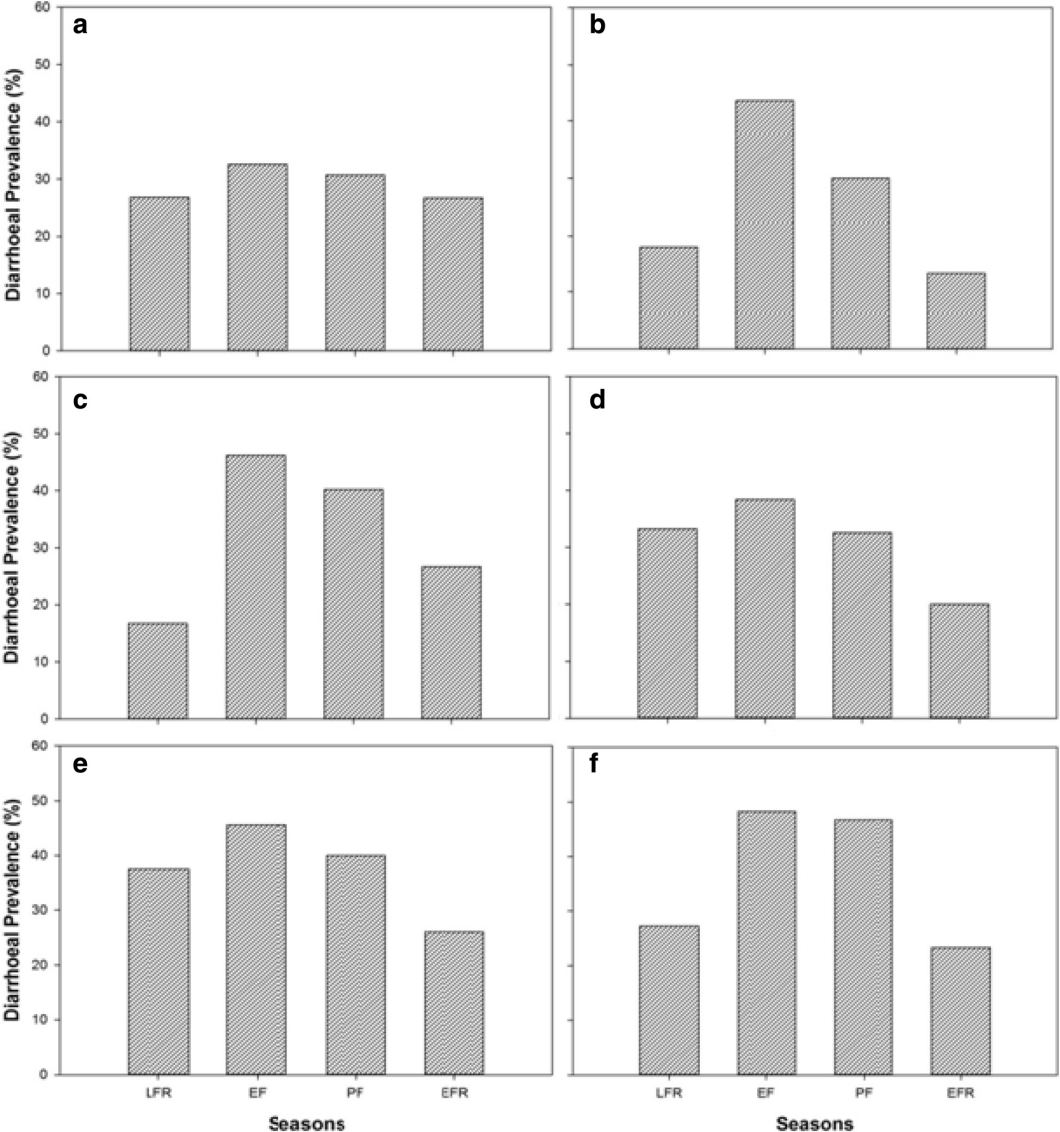
Temporal variation of households’ diarrheal encounters within settlements of **a** Boro, **b** Maun, **c** Xobe, **d** Samedupi, **e** Chanoga, and **f** Motopi along the Boro-Thamalakane-Boteti river system. Seasons *LFR* late flooding recession, *EF* early flooding, *PF* peak flooding, *EFR* early flood recession

Comparisons of households’ diarrheal encounters for different sampled seasons using ANOVA revealed a marked variation in diarrheal prevalence during the study period (F _(3, 20)_ = 11.02, *p* = 0.000). Post hoc comparisons showed that prevalence during the early flooding season (May) was significantly higher than the flood recession seasons (February (*p* = 0.03) and December (*p* = 0.000)) but less than during the peak flooding season (September). Diarrhea cases during peak flooding season (September) was significantly higher than during late flood 41 recession season (December, *p* = 0.007) (Fig. 7a). Diarrhea cases during the peak and flood recession seasons was not significantly different (*p* > 0.05). Even though Boro and Maun tended to record slightly less diarrhea cases than downstream settlements of Xobe, Samedupi, Chanoga, and Motopi during different seasons (Fig. 7b), spatial variation was not statistically significant (F _(5, 18)_ = 0.555, *p* = 0.733).

### Households’ diarrheal encounters and water use practices

The practice of boiling water for domestic purposes was a significant predictor of households’ diarrheal encounters (*p* = 0.028). However, there was no significant relationship between prevalence of diarrhea and whether residents used river water for all household purposes including drinking (Table 3). There was no significant association between the number of households with standpipes and households’ diarrheal encounters in the study settlements (*p* > 0.05).

**Table 3:** Multiple regression coefficients where households’ diarrheal encounters was the dependent variable

Independent variables	Coefficient	Standard error	*t* value	*p* value
RW treated before use	0.643	0.162	3.967	0.028^a^
RW used for drinking	0.007	0.067	0.108	0.921

*RW* river water

^a^Significant

### Households’ diarrheal encounters and river water quality

*E. coli* and fecal streptococci concentrations were found to be significant predictors (*p* = 0.0394 and *p* = 0.040, respectively) of households’ diarrheal encounters only during the early flooding season around May, while turbidity was a significant (*p* = 0.045) predictor of household diarrheal encounters during the peak flooding season in September (Table 4). The conductivity, pH, and dissolved oxygen did not show any significant relationship (*p* > 0.05) between water quality and households’ diarrheal encounters in the study settlements.

**Table 4:** Multiple regression coefficients of selected water quality parameters during various seasons where households’ diarrheal encounters was the dependent variable

	Seasons											
Independent variables	LFR			EF			PF			EFR		
	(*β*)	*t*	*p*	(*β*)	*t*	*p*	(*β*)	*t*	*p*	(*β*)	*t*	*p*
	*R* ^2^ = 0.559			*R* ^2^ = 0.953			*R* ^2^ = 0.930			*R* ^2^ = 0.857		
*Escherichia coli*	−0.118	−0.241	0.832	0.451	4.865	0.0394**	−0.042	−1.089	0.390	−0.164	−2.558	0.1249
Fecal streptococci	−0.033	−0.154	0.982	−0.960	−4.825	0.040^a^	−0.0343	−0.446	0.699	−0.0352	−0.387	0.736
Turbidity	−0.0737	−0.167	0.883	−0.343	−0.803	0.506	2.037	4.536	0.0453^a^	1.144	1.338	0.313

Seasons—*LFR* late flooding recession, *EF* early flooding, *PF* peak flooding, *EFR* early flood recession, *β* regression coefficient

^**^Significant

## Discussion

Many communities worldwide are dependent on rivers in their vicinity for livelihoods despite high pollution of such water sources [28–30]. Similarly, we observed high dependency on the contaminated Boro-Thamalakane-Boteti river system for domestic purposes, even in the gazetted settlements of Maun, Chanoga, and Motopi which are supposedly provided with treated piped water by the government through the Department of Water Affairs and Water Utilities with treated water supplies. Lack of treated piped water supplies explains high household dependency on river water in ungazetted settlements of Xobe and Samedupi. In contrast, residents in gazetted settlements of Boro, Maun, Chanoga, and Motopi have been compelled to use untreated surface water from the Boro-Thamalakane-Boteti river system by the unreliability and unpredictability and poor taste (due to high salinity, color, and microbial concentrations) of the piped water supply [31]. Furthermore, 51 % of households in Maun reported acute water shortage and no water flow from pipes [32].

Furthermore, very few communal standpipes have been reported to be functional in Maun and other settlements due to frequent breakdowns of boreholes caused by floods and lack of spares [30]. Tubatsi et al. (2014) [25] reported that although the surface water is not saline (i.e., its freshwater), it was also unsuitable for domestic use as it contained high *E. coli* and fecal streptococci concentrations and turbidity levels which exceeded the Botswana Bureau of Standards recommended limits for drinking water. The high *E. coli* and fecal streptococci concentrations in the river water are probably from fecal contamination by animals since most people in the studied settlements using the same water sources for watering their livestock and wild animals [31].

Even though the use of polluted surface water sources has been implicated for diarrheal outbreaks in other studies [28, 29, 33], our results seem to suggest that river water quality at source may not be the only predictor of households’ diarrheal encounters in our study settlements. Although some respondents interviewed by Kaluli et al. (2011) [34] associated diarrhea and skin rash diseases with use of untreated surface water in settlements around the Okavango Delta, we were unable to demonstrate any significant relationship between water quality in the Boro-Thamalakane-Boteti river system and households’ diarrheal encounters in our study settlements during the LFR, PF, and EFR seasons. River water quality was only significantly related to households’ diarrheal encounters during the early flooding (EF) season around May when *E. coli* and fecal streptococci counts in the river water (CFU/100 ml) and households’ diarrhea cases in the study settlements were highest. A similar lack of association between river water quality and diarrheal prevalence has previously been reported in Ethiopia [33]. Perhaps the pollution of water in the river system during the rest of the seasons was moderate enough not to pose a significant health risk as argued by [35] who advocated for a threshold effect of indicator density for diarrheal risk.

Even though isolation of rotavirus was beyond the scope of this study, it might have been the main etiological agent responsible for the highest diarrheal prevalence during cold and dry early flooding season (May). Rotavirus, which has been associated with most diarrheal outbreaks in the sub Sahara region [36–38], occurs mostly during cold dry seasons of the year [34, 36, 39].

Results are suggestive of other possible diarrheal risk factors other than source water quality. Even though some have argued that post-source contamination present little and insignificant health risk [40, 41], our results question the strength of this argument. Lending support from previous research where unhygienic storage conditions were underlined to contribute to declining of water quality in households [40–43], we argue that the unhygienic storage conditions observed during the survey, such as inadequate care for and washing of storage containers and storage vessels with no lids or lids not fitting properly, might have possibly led to further decline of water quality in households. Further contamination might have also occurred during transportation from water source as most surveyed households lacked on-plot water connections. On a similar note, Hamer et al. (1998) [44] positively correlated water availability in a household with good hygiene behavior while [45] associated distance from house to source with diarrhea.

This study also reaffirms the benefits of boiling water in households. As expected, settlements where more households boiled water experienced significantly less diarrhea prevalence than their counterparts. Studies elsewhere have shown that adoption of measures such as boiling to improve drinking water quality at home effectively reduces diarrheal risks [20, 21, 45]. The high boiling temperatures denatures bacterial proteins needed to function and reproduce, rendering the bacteria non-viable and making the water safer.

We acknowledge the complexity of factors driving prevalence of diarrhea making prediction of their contribution to a high degree of accuracy relatively difficult. This study only focused on water use practices and water quality, but other factors such as sanitation, individuals’ natural immunity, HIV/AIDS, malnutrition, and other infections can limit individuals’ response to diarrhea [37]. As put forward by Trevett et al. (2004) [39], it is also possible that these communities’ farming activities such as animal keeping contributed to some cases of diarrhea. Some residents were observed bathing in the river, an activity which has been linked with diarrhea in other studies [46–47] and could have possibly contributed to some diarrhea cases in communities along the Boro-Thamalakane-Boteti river system. Further research is therefore necessary to elucidate other factors. Further research can also be done to evaluate the effectiveness and adoption of different water treatment techniques.

## Conclusions

This study has provided evidence that although provision of safe water is essential for reducing diarrheal burden, as a single effort, it is not sufficient. The quality of water and how the water is stored at home may contribute to increased diarrheal diseases burden. Therefore, integrated control programs focusing on improving quality of water both at source and point of use will be more effective. Specifically, promotion of good hygiene practices is essential.
